# Leveraging artificial intelligence for prediction of pulmonary hemorrhage in preterm infants

**DOI:** 10.1038/s41372-025-02390-2

**Published:** 2025-08-20

**Authors:** Hany Aly, Vanishree Nandakumar, Hasan Cetin, Hatem Eltaly, Tetyana Nesterenko, Mohamed A. Mohamed

**Affiliations:** 1 Division of Neonatology, Cleveland Clinic, Cleveland, OH USA; 2 Cleveland Clinic Foundation, Cleveland, OH USA; 3 Division of Neonatal-Perinatal Medicine, University of Texas Southwestern Medical Center, Dallas, TX USA

**Keywords:** Risk factors, Biomarkers

## Abstract

**Objectives:**

To identify clinical variables and indicators associated with pulmonary hemorrhage in preterm infants.

**Methods:**

This case-control study included inborn infants <32 weeks. Data were collected in 12-h epochs from birth until hemorrhage onset or up to 72 h for controls. Machine learning used the Random Forest algorithm. Statistical analysis included *T* test and Mann–Whitney U test.

**Results:**

Among 1133 screened infants, 35 had hemorrhage. Mean gestational age was 25.6 ± 1.6 weeks, birth weight 753 ± 224 g, and median onset of hemorrhage was 44.5 h. Affected infants more often required chest compressions and invasive ventilation. Machine learning (accuracy = 83%, AUC = 90%) identified repeated surfactant dosing and postnatal hypotension in the first 12 h of life as top predictors, along with maternal and gestational age. Mortality was higher in cases than controls (19% vs. 3%, *p* = 0.005).

**Conclusion:**

Repeated surfactant dosing and early postnatal hypotension are key predictors for pulmonary hemorrhage in preterm infants.

## Introduction

Preterm infants are at risk of several morbidities, of which one of the most acute complications occurring within the first few days of life is pulmonary hemorrhage (PHEM). PHEM is characterized by disruption of pulmonary vascular endothelial barrier leading to extravasation of blood into the pulmonary alveolar space. Infants with the lowest birth weight and youngest gestational age are the highest risk of developing PHEM [1]. PHEM is not common; it occurs in <10% of preterm infants. However, its associated mortality is quite high at 50–68% [2, 3]. Among the survivors, bronchopulmonary dysplasia (BPD) and neurodevelopmental abnormalities are commonly seen [4].

There are multiple risk factors that predispose to PHEM such as hyaline membrane disease, mechanical ventilation, surfactant administration, patent ductus arteriosus (PDA), thrombocytopenia, bleeding disorders, and coagulopathies [4]. These risk factors are not specific, as all preterm infants encounter many of these factors. Therefore, there is unmet need to identify specific predictors or markers of PHEM in preterm infants. Once the predictors are identified and mitigated, PHEM can potentially be avoided.

Healthcare digitalization has facilitated electronic monitoring, record keeping, and data management in neonatal intensive care units (NICUs). Artificial intelligence (AI) programs have recently analyzed electronic data to predict several neonatal complications [5, 6]. These models have been increasingly utilized for prediction, image interpretation, clinical decision making and improving neonatal outcomes. There has been no study that utilized machine learning to predict the development of PHEM. With this study, we aimed to propose an artificial intelligence model based on machine learning to identify modifiable variables that would predict pulmonary hemorrhage in preterm infants. The aims of this study were to: (1) assess modifiable variables associated with PHEM utilizing traditional statistical analyses, (2) in parallel, use a machine learning model to predict infants developing PHEM, and (3) reconcile the AI findings with statistical findings. We hypothesized that variables identified by statistical analyses would not differ from those provided by AI.

## Patients and methods

### Setting

This retrospective study was conducted at Cleveland Clinic Children’s Hospital that has two level 3 and one level 4 neonatal intensive care units (NICUs) with approximately 10,000 deliveries annually.

### Patients

The study included all infants with gestational age (GA) < 32 weeks and/or birth weight (BW) < 1500 g with a documented diagnosis of PHEM. For each case with PHEM, 2 controls were selected who were born in the same year, and were chronologically closest to the case, had the same week of GA, and had the BW within 50 g of the case. The study utilized data from January 1st, 2013, through December 31st, 2021. Hemorrhage was diagnosed if profuse bleeding was discovered in the airways that required significant escalation of respiratory support. Infants with blood-tinged secretions in the endotracheal tubes were not diagnosed with PHEM. Given the pilot nature of the study and the sparce nature of the disease, power analysis was not applicable. However, the study included all available cases with PHEM over 9 years.

### Delivery room management

Delivery rooms were staffed by maternal-fetal medicine specialists and in-house neonatologists. Delayed cord clamping (>30 s) was routinely practiced for all deliveries, unless deemed unfeasible. A change in neonatal resuscitation practices occurred during the study period. Prior to 2019, all extreme preterm infants were routinely intubated and administered surfactant. After 2019, guidelines were revised to favor initial resuscitation with continuous positive airway pressure (CPAP) in the delivery room, except for periviable non-vigorous preterm, followed by bubble CPAP upon admission to the NICU. No other significant changes were recalled regarding the use of prenatal steroids, magnesium sulfate, or postnatal caffeine.

### Data management

EMR was retrieved, and multiple maternal and neonatal variables were collected. Maternal data included age, race, parity, the presence of gestational hypertension, the presence of chorioamnionitis, platelets count on the day of delivery, timing of amniotic membrane rupture, antepartum steroid administration, and the use of magnesium prophylaxis. Infants’ data collected included Apgar scores at 1, and 5 min, sex, BW, mode of birth, and delivery room resuscitation measures (intubation, chest compression, and use of epinephrine). Clinical and laboratory data were collected in 12-h epochs for the first 72 h or until PHEM occurred. The data in each epoch included three values for each of the following variables: heart rate, blood pressure, fraction of inspired oxygen (FiO_2_), and measured blood gas components. The three values represented the mean, the lowest, and the highest values during each 12-h period. Mode of ventilation, complete blood count, surfactant administration, fluid intake and use of caffein were all recorded.

### Statistical analysis

Continuous variables were summarized with mean (M) and standard deviation (SD) or medians (MED) and interquartile ranges (IQR) and compared using *T* test or the Mann–Whitney U test. Categorical variables were reported with frequencies and percentages and compared using the chi-squared tests. A *p* value < 0.05 was considered significant.

#### AI model training

##### Data processing

Data pre-processing involved encoding categorical variables, handling missing values, and normalizing continuous variables to ensure model compatibility. Infants were stratified into categories based on BW (<700, 700–1000, and >1000 g) and GA (<25 weeks, 25–27 weeks, and >27 weeks). Further, to achieve maximum accuracy, we eliminated infants with missing data for training the AI model. Continuous variables were entered in the model after being categorized into nominal variables. For example, arterial blood pressure values within each epoch were categorized into 3 categories: 0,1 & 2. These categories were assigned based on the lowest quartile, middle half and highest quartile of all values. Consequently, within each 12-h epoch, blood pressure in the lowest 25%ile would receive a score of 0, whereas the highest 25%ile readings and the mean may receive scores of 2 or 1 based on the actual blood pressure measurement. Similar approaches were charted for heart rate, oxygen requirement, and blood gas parameters. Of note, these scores were generated solely for the purposes of the model and did not necessarily reflect clinical diagnoses such as hypotension, tachycardia, or acidemia. A list of categorized variables is provided in the supplemental table.

##### Model training

A random forest algorithm was employed for model training due to its robustness, ability to handle both categorical and continuous variables, and strong performance in medical prediction tasks. The Random Forest algorithm is an ensemble machine learning method that builds multiple decision trees during training and aggregates their results to improve prediction accuracy and generalizability [7]. Each tree is trained on a random subset of the data and a random subset of features, which helps reduce overfitting and variance. This approach can be likened to consulting multiple independent experts—each providing an opinion based on different evidence—and then combining their responses to reach a consensus, which often yields a more reliable decision than relying on a single opinion. Each “expert” is actually a decision tree. A forest is a collection of these decision trees. The trees are built randomly and independently, hence the name: Random Forest.

Random Forest was selected for this study due to its robustness in handling high-dimensional data, its ability to model complex, non-linear relationships, and its resistance to overfitting and outliers [7, 8]. Moreover, it provides useful measures of feature importance, which are valuable for interpreting model results. These characteristics make Random Forest a suitable and effective choice for our dataset, which includes diverse and potentially noisy features.

In this study, the model was trained on 90 patient records using 80 decision trees (estimators), with each tree allowed a maximum depth of 15 decision nodes, a parameter that controls model complexity and helps avoid overfitting. For example, in predicting respiratory distress, one decision tree might split data based on gestational age, birth weight, and Apgar score, while others use different combinations. To ensure reliability, we applied 10-fold cross-validation within the training dataset. This technique partitions the data into 10 subsets, using nine for training and one for validation, rotating through all subsets. This improves generalizability and minimizes the risk of overfitting.

##### Model evaluation

The performance of the trained model was evaluated using a separate holdout test set, ensuring an unbiased evaluation of the model’s predictive power in real-world scenarios. Model discrimination was evaluated using the area under the receiver operating characteristic curve (AUC-ROC), while calibration—the agreement between predicted probabilities and observed outcomes—was assessed using a calibration plot. The calibration curve was generated by plotting predicted probabilities against observed outcome frequencies using a LOESS smoothing function, with a 45-degree reference line indicating perfect calibration. Calibration slope and intercept were also calculated to assess model fit. Internal validation was performed using cross-validation within the training dataset to reduce the risk of overfitting.

Lift curve construction and interpretation: Patients were ranked in descending order by their predicted probability of pulmonary hemorrhage. The lift curve plots the cumulative proportion of patients flagged (x-axis) against the proportion of true positive cases captured (y-axis), following the approach described by Powers (2015) [9]. This ordering ensures that the curve reflects the model’s ability to prioritize high-risk cases. Area under the precision-recall curve (AUPRC) and related metrics were also assessed.

In this case–control design, hemorrhage prevalence was 33% (1 case per 2 controls). Therefore, randomly selecting 33% of patients would yield 33% of true positives by chance. This diagonal “baseline” or “chance” line in the lift plot represents the expected performance of a non-informative model. Our model’s performance exceeds this baseline—at the 0.50 cutoff, for example, it captures ~71% of true positives compared to 33% expected by chance (lift = 2.1).

## Results

Of the 1133 premature infants reviewed, 35 (3%) infants had PHEM. All of them were included in the study. A total of 70 matched controls were included. The cases had mean GA of 25.6 ± 1.6 weeks and BW of 753 ± 224 g. Most cases were intubated in the delivery room (86%), and nine of them (26%) received chest compressions. Maternal characteristics, mode of delivery, and delivery room resuscitation did not differ between cases and controls, Table 1.

**Table 1:** Maternal, infant and neonatal management in cases and controls (*n* = 105).

	Cases (*N* = 35)	Controls (*N* = 70)	*P* value
**Maternal variables**			
Maternal age^a^	27.9 ± 4.9	27.9 ± 6.1	0.98
Prenatal steroids	35 (100)	70 (100)	NS
Prenatal magnesium sulfate	30 (86)	59 (84)	0.84
Maternal platelets count (x1000)^a^	222 ± 76	218 ± 70	0.81
PPROM, n (%)	3 (8.5)	17 (24.2)	0.057
Chorioamnionitis, n (%)	4 (11.4)	14 (19.4)	0.28
Mode of delivery (Cesarian), n (%)	24 (69.4)	53 (76.1)	0.50
**Infants variables**			
Gestational age, weeks^a^	25.6 ± 1.6	25.6 ± 1.5	0.96
Birth weight, grams^a^	753 ± 224	747 ± 216	0.90
Small for gestational age, n (%)	6 (16.7)	11 (16.4)	0.97
Male sex, n (%)	26 (74)	40 (57)	0.07
Race/ethnicity			
Caucasian	13 (37)	30 (43)	
African American	12 (34)	21 (30)	0.84
Hispanic & Others	10 (29)	19 (27)	
Apgar score at 1 min^b^	3 (4)	3 (4)	0.4
Apgar score at 5 min^b^	6 (5)	7 (5)	0.16
**Neonatal management & outcomes**			
DR intubation, n (%)	31 (86.1)	51 (72.8)	0.052
DR chest compression, n (%)	9 (25)	7 (10.4)	0.03
DR epinephrine, n (%)	4 (11.1)	2 (3.0)	0.07
Surfactant 1, n (%)	35 (97.2)	64 (91.4)	0.054
Surfactant 2, n (%)	31 (88.6)	38(54.2)	0.002
Surfactant 3, n (%)	23 (63.9)	10 (14.9)	0.0017
Caffeine on admission	34 (97.1)	61 (87.1)	0.74
Fluid intake, ml/kg/day^a^			
Day 1	98 ± 33.3	100.2 ± 23	0.69
Day 2	107.9 ± 29.1	127.4 ± 21.4	0.001
Day 3	136.4 ± 44.1	153.7 ± 31.7	0.07
Intorope use	8 (23)	4 (10)	0.02
Mortality, n (%)	7 (19.4)	4 (5.7)	0.02
Bilateral IVH	13 (36.1)	13 (18.5)	0.06
Severe IVH (Grades III & IV)	8 (22.2)	5 (7.14)	0.04

Data are expressed in numbers (%) chi square test was used, except with ^a^ data expressed in mean ± SD and *t* test was used and with ^b^ data expressed in median (IQR) and Mann–Whytney test was used.

*DR* delivery room, *IVH* intraventricular hemorrhage, *PPROM* preterm premature rupture of membranes.

The median PHEM onset was at 44.5 h of life (Interquartile range (IQR = 34–120)). Infants in PHEM group were more likely to receive more than a single dose of surfactant replacement therapy (89% vs 55%, *p* = 0.002). They were more likely to be supported with invasive mechanical ventilation during the six 12-h epochs studied. A total of 27 infants in the PHEM group was diagnosed with PDA of which 17 of them received medical management with either indomethacin (*n* = 6), ibuprofen (*n* = 2) or acetaminophen (*n* = 9). Incidence of bilateral intraventricular hemorrhage was significantly higher in the PHEM group compared to controls (36% vs 25%, *p* = 0.014). The mortality was significantly higher in the PHEM group (19% vs. 3%, *p* = 0.005).

### AI model performance

**Fig. 1:**
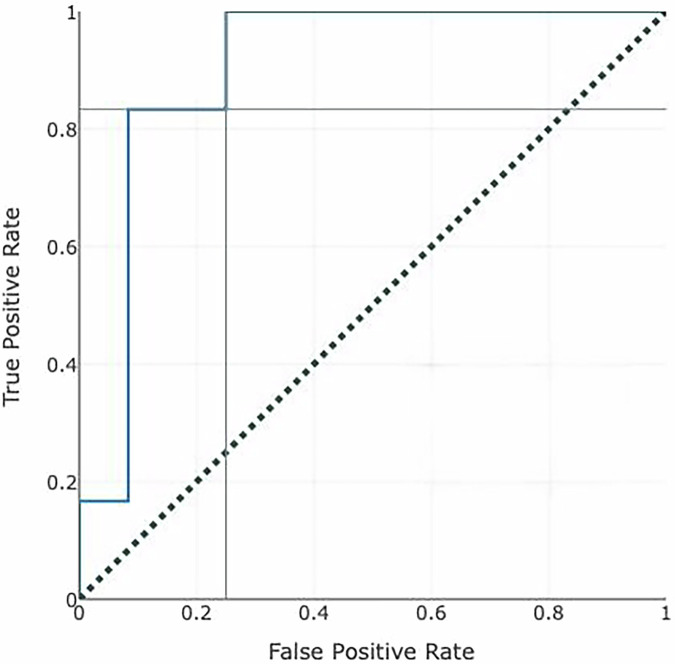
Receiver operating characteristic (ROC) for prediction of pulmonary hemorrhage in premature infants. Area under the curve = 0.90 (95% CI: 0.83–0.97), Cut off = 0.50, true positive rate = 0.83, and false positive rate = 0.25.

**Fig. 2:**
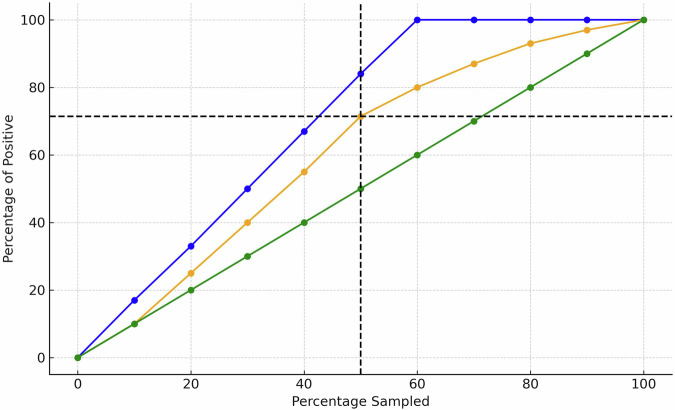
Lift curve for predicting pulmonary hemorrhage in premature infants. The lift curve compares the performance of the predictive model for pulmonary hemorrhage in premature infants against a perfect model and random selection. The x-axis represents the percentage of the population sampled, while the y-axis indicates the percentage of positive cases identified. The upper blue line represents a perfect classifier, the middle orange line represents the model’s performance, and the lower green line corresponds to a random classifier. At a cutoff of 0.50, the model captures 71.43% of true positive cases compared to 33.33% expected by chance, yielding a lift of 2.1.

**Fig. 3:**
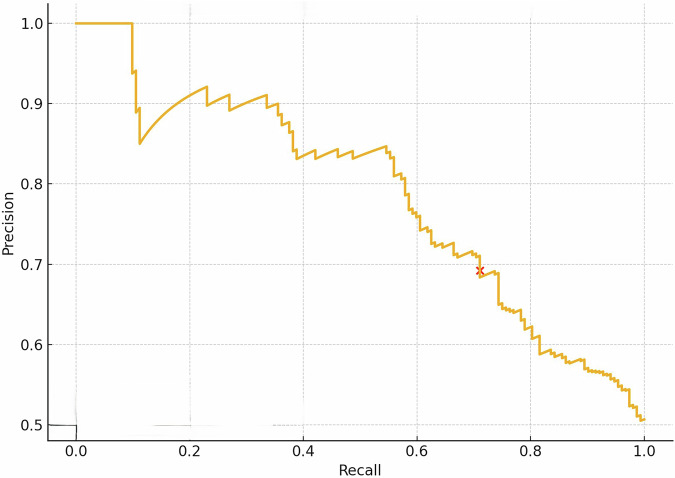
Precision–recall curve used to assess model performance. The area under the precision recall curve is 0.79. At the standard 0.50 cutoff, the model achieved a precision of 0.69, recall of 0.71, and an F1 score of 0.70.

**Fig. 4:**
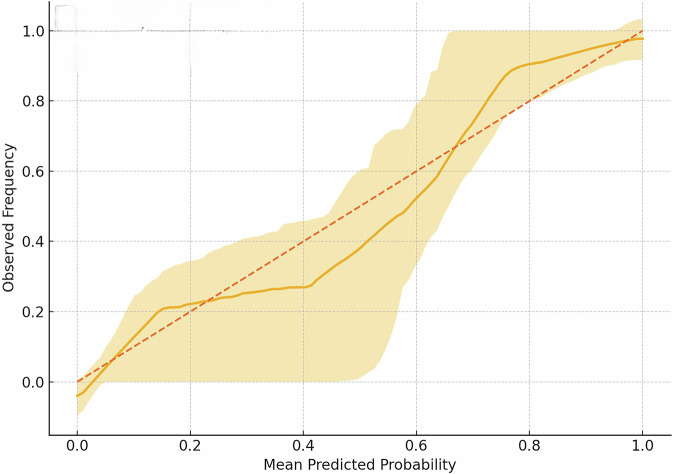
The calibration of the random‐forest model. Using LOESS smoothing (gold line) with 95% bootstrap confidence intervals (shaded). The calibration curve closely follows the diagonal, indicating good agreement between predicted probabilities and observed pulmonary hemorrhage rates across deciles of risk.

Using the random forest model, a strong predictive performance was demonstrated and was maintained during the ten -time cross-validation phases, with an average accuracy of 83% and an area under the ROC curve (AUC) of 0.90 with 95% CI: 0.83–0.97 (Fig. 1). On the holdout test set, the accuracy of the model was shown at 71% with an AUC of 0.83 (Fig. 2). The prediction scores ranged from 0.65 to 0.73, indicating the confidence in predicting the occurrence of PHEM. We assessed model performance using the precision–recall curve, yielding an AUPRC of 0.79 (Fig. 3). At the standard 0.50 cutoff, the model achieved a precision of 0.69, recall of 0.71, and an F1 score of 0.70. Figure 4 shows the calibration of our random‐forest model using LOESS smoothing with 95% bootstrap confidence intervals. The calibration curve closely follows the diagonal, indicating good agreement between predicted probabilities and observed pulmonary hemorrhage rates across deciles of risk.

**Fig. 5:**
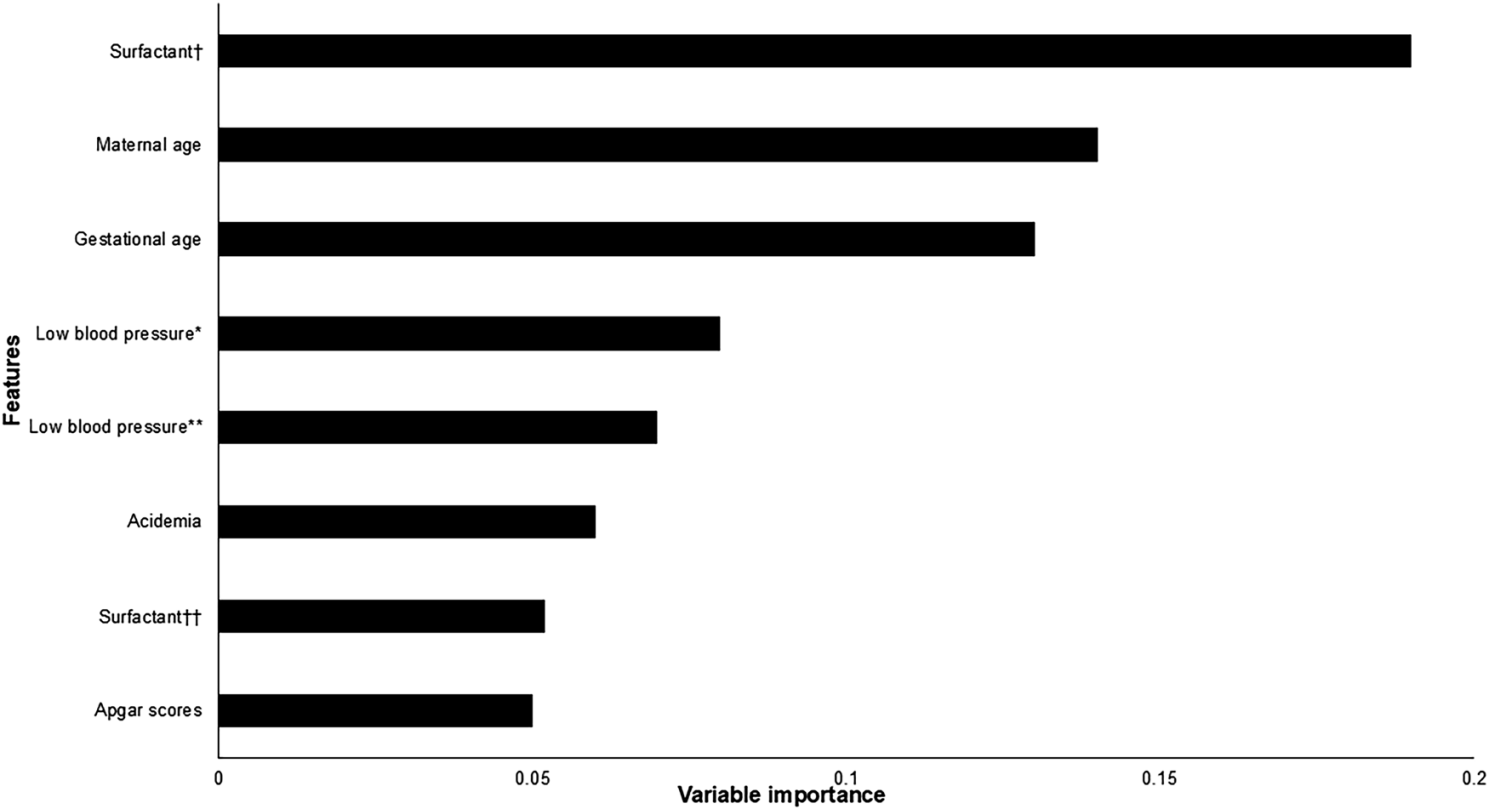
Feature importance in predicting pulmonary hemorrhage in premature infants. This feature importance plot illustrates the relative contribution of various clinical factors in predicting pulmonary hemorrhage in premature infants. The x-axis represents variable importance, while the y-axis lists the predictive features. Higher values indicate greater influence on the model’s predictions. The most important features include repeated dosing of surfactant, maternal age, and gestational age, followed by low blood pressure parameters and acid-base status. †Three doses of surfactant; †† Two doses of surfactant; * Low blood pressure 0–12 h low (values < 22 mmHg) vs. intermediate (values 22–29 mmHg); ** Low blood pressure 0–12 h low (values < 22 mmHg) vs. high (value > 29 mmHg).

Analysis for predictive factors showed repeated dosing of surfactant, postnatal hypotension, and acidemia as the strongest predictors for the development of PHEM. Non-modifiable factors included maternal age and gestational age (Fig. 5).

**Fig. 6:**
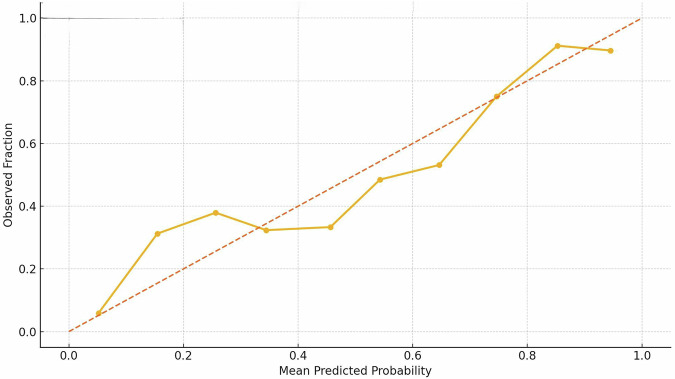
Calibration curve for predictive model. The solid line represents model calibration, and the dotted line represents perfect calibration.

Calibration was assessed using a calibration plot, which demonstrated good agreement between predicted and observed probabilities. The calibration slope was 0.97 and the intercept was −0.02, indicating minimal overfitting and no major systematic bias in risk estimation (Fig. 6).

The model’s prediction scores ranged from 0.65 to 0.73, indicating moderate to high confidence in the prediction of PHEM events. Feature importance analysis identified repeated dosing of surfactant, postnatal hypotension, and acidemia as the strongest predictors for PHEM. Non-modifiable factors such as maternal age and gestational age were also associated with increased risk (Fig. 5).

**Fig. 7:**
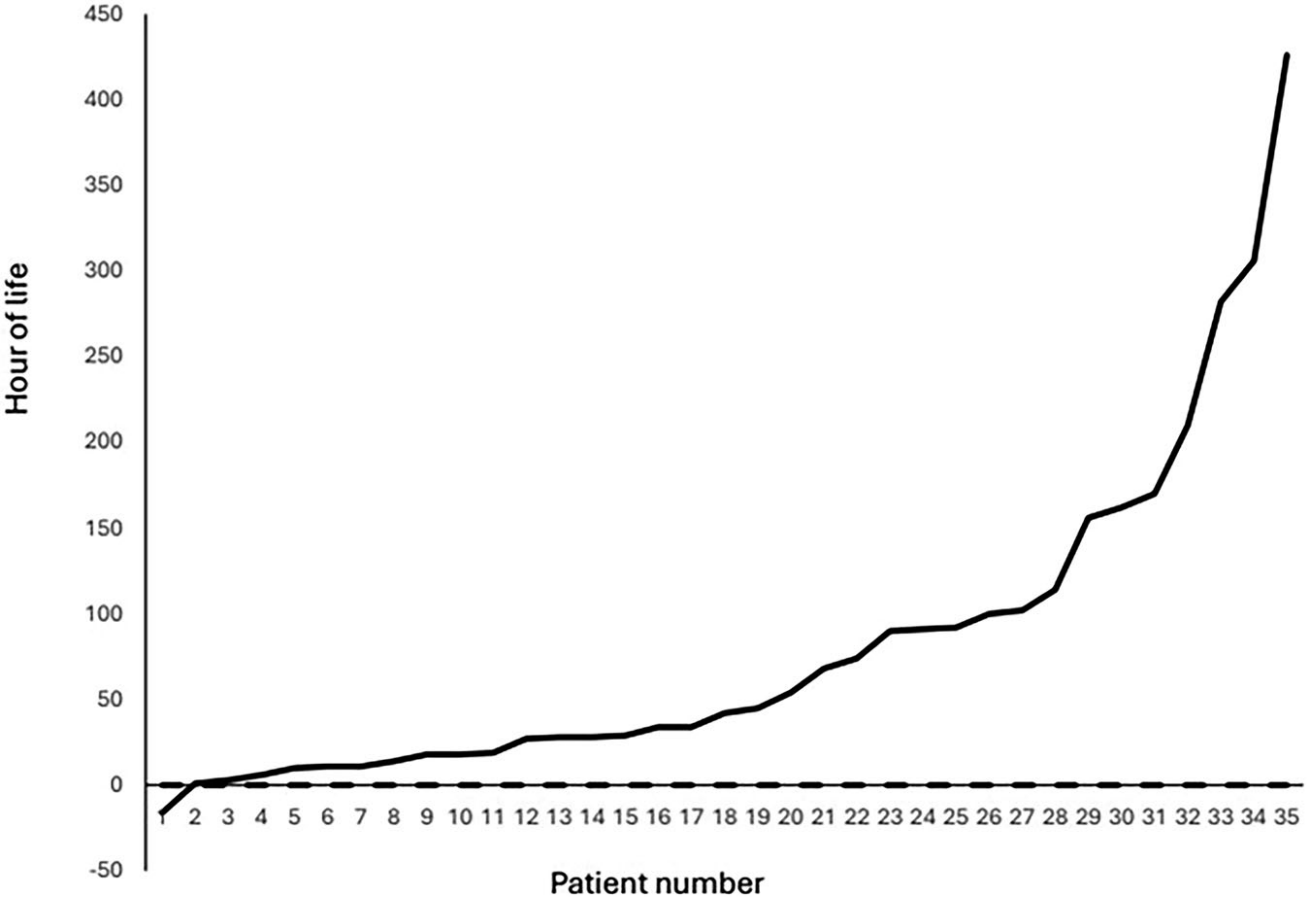
Duration between blood pressure nadir and pulmonary hemorrhage in premature infants. This figure illustrates the duration between the lowest recorded blood pressure and the occurrence of pulmonary hemorrhage in premature infants. Understanding this time lag is critical for identifying potential windows for intervention. The distribution of time intervals highlights patterns that may guide clinical decision-making and early therapeutic strategies to mitigate the risk of pulmonary hemorrhage.

To revalidate the findings of AI modeling, we compared the mean blood pressure in cases vs controls in each 12-h epoch. Mean arterial blood pressure did not differ between cases and controls during any of the 12-h study epochs of the first 72 h of life. We further explored the temporal relationship between lower blood pressure events and the diagnosis of PHEM. In each case with PHEM, we identified the duration between the nadir of the mean blood pressure and the incidence of PHEM. Among the 35 infants with PHEM, the majority of if infants had their lowest blood pressure in the first 12 h of life (median age 6 h). The median lag between the nadir of blood pressure and the later onset of PHEM was 42 h (mean 82 h) (Fig. 7).

Fluid management was generally more conservative in cases than controls. Fluid intakes (mL/kg/d) in cases and controls were 98 ± 33.3 vs 100.2 ± 23 (*p* = 0.69), 107.9 ± 29.1 vs 127.4 ± 21.4 (*p* = 0.0007), and 136.4 ± 44.1 vs 153.7 ± 31.7 (*p* = 0.07) in days 1, 2, & 3, respectively. Inotropes were more frequently utilized in cases than controls (23% vs 10%, *p* = 0.02).

## Discussion

In this study, we investigated predictors of pulmonary hemorrhage (PHEM) in premature infants using both traditional statistical analyses and advanced machine learning (ML) models. The findings indicate that repeated dosing of surfactant and postnatal hypotension are among the strongest predictors of PHEM, while nonmodifiable factors such as lower gestational age and maternal age also contribute to prediction. Notably, using AI-driven model, the study achieved an AUC of 90% during cross-validation and 83% on the holdout test set, underscoring the potential of AI-based predictive modeling for the early identification of high-risk infants.

Hypotension was associated with PHEM. Whether this hypotension was attributed to the steal phenomenon when blood shunted from the aorta across the ductus arteriosus is not clear. Of note, most premature infants maintain their ductus arteriosus open early in life, and clinical trials aimed at closing PDA have not demonstrated a decreased prevalence of PHEM [10]. This suggests that hypotension associated with PDA, rather than PDA itself, is associated with PHEM. Acidemia was also associated with PHEM, aligning with its connection to hypotension. When hypotension becomes severe enough to cause hypoperfusion and metabolic acidemia, it likely serves as the trigger for microvascular injury and subsequent PHEM. Notably, carbon dioxide concentrations in blood gases did not differ between cases and controls, indicating that the acidemia observed in this study was metabolic in origin. Surfactant replacement remains the standard therapy for intubated infants with respiratory distress syndrome. However, this study found that repeated surfactant dosing was associated with the development of PHEM. A known mechanism linking improved lung compliance to increased pulmonary congestion—attributed to higher PDA flow—could explain this association. The enhanced pulmonary circulation following surfactant administration may contribute to hemodynamic instability and the extreme hypotension reported in cases of PHEM [11].

There are notable similarities in the pathogenesis of PHEM and intraventricular hemorrhage (IVH), both of which occur primarily in premature infants. Both conditions are linked to vascular fragility, impaired autoregulation in the brain and lungs, and hemodynamic instability. Previous studies have identified hypotension-induced reperfusion injury as a primary cause of IVH in premature infants [12], further strengthening the claim of the possible role of hemodynamic disturbances in PHEM development.

Analysis of hemodynamic data further detailed the of the timing of blood pressure dynamics and the later development of PHEM. As illustrated in Fig. 5, the median time between the onset of early hypotensive events (approximately 6 h of life) and the subsequent development of PHEM (median onset: 44.5 h). Figure 3 complements these findings by demonstrating that more profound drops in blood pressure are associated with hemorrhagic outcomes. It is important to emphasize that, due to the study’s observational design, only associations—not causality—can be inferred between hypotension and the development of PHEM. Infants with PHEM received less fluid and more inotropes compared to controls, suggesting that the observed association is unlikely to be explained by aggressive fluid resuscitation. The potential role of inotrope use in PHEM warrants further investigation. However, unmeasured confounders related to hypotension may have influenced the results and were not accounted for in the analysis.

Blood pressure for each subject was assigned a score based on the quartile in which the measurement fell within the cohort, rather than on bedside clinical diagnosis or established normative values. Notably, the definition and management of hypotension in premature infants remain unsettled. While some clinicians advocate for aggressive treatment, others adopt a permissive approach. Nonetheless, this study offers a novel perspective on a potential association that warrants further investigation.

A key novel aspect of the current study is the comparative analysis between traditional statistical methods and AI-based prediction. While conventional analyses identified significant predictors, —including repeated surfactant dosing—the study captured, via ML model, complex, nonlinear relationships among clinical variables that were not evident through standard methods. For instance, traditional approaches did not detect significant differences in arterial blood pressure between cases and controls when analyzed over 12-h epochs, yet using the AI model, postnatal hypotension was consistently flagged as a robust predictive factor. This discrepancy underscores the ability of utilizing AI to uncover subtle temporal interactions and patterns that may be overlooked by classical statistical techniques.

The temporal association observed in the current study is particularly noteworthy. The lag between early hypotensive episodes and later PHEM suggests that hypotension may predispose to pulmonary vascular instability, rather than being a simultaneous occurrence with hemorrhage. This insight not only reinforces the need for vigilant hemodynamic monitoring in the NICU but also highlights a potential period for early intervention that could mitigate the progression to severe PHEM.

The current study benefits from a well-defined case-control design, comprehensive electronic medical record data, and rigorous validation of AI predictions. However, several limitations must be acknowledged. The retrospective design inherently carries risks of bias due to missing data and selection criteria. Observational design allows only for assessment of association and not causation. The study was conducted at two sites only that would limit generalizability of findings. Moreover, although high predictive accuracy was noted via AI model, its generalizability to external populations requires further validation. Finally, the interpretability challenges associated with AI models remain an area for future improvement to facilitate their integration into clinical practice. PHEM is infrequently encountered in premature infants, therefore, the number of cases enrolled in this analysis was understandably small. However, the current study provides proof of concept for future studies involving multiple centers. All infants in this study were initially intended to receive aggressive resuscitation, as determined through collaborative decision-making between caregivers and parents. In some cases, care may have been redirected following uncontrollable hemorrhage and a determination that further intervention was futile. The study did not distinguish between infants who died following full resuscitation and those whose care was redirected. However, this distinction is unlikely to influence the primary outcome of interest.

In conclusion, our integrated analysis identifies repeated surfactant administration and postnatal hypotension as key predictors of PHEM in preterm infants. The observed temporal lag between early hypotensive events and the onset of hemorrhage provides a promising window for early clinical intervention. Moreover, the superior performance of the used AI model in PHEM prediction—by capturing complex interactions that conventional analyses may miss—offers a compelling argument for the adoption of advanced analytical methods in neonatal care. These findings lay the groundwork for developing a large multicenter cohort study to validate the findings of the current study before any intervention can be proposed. Future research should focus on the prospective validation of these predictive models and the integration of real-time predictive tools into NICU workflows.

## Supplementary information

Supplemental Table

## Data Availability

The datasets generated and/or analyzed during the current study are available from the corresponding author on reasonable request.
