# Supplementary material for: Leveraging artificial intelligence for prediction of pulmonary hemorrhage in preterm infants
Source: J Perinatol. 2025 Aug 20;45(10):1403–10. doi: 10.1038/s41372-025-02390-2 (PMC12479356; doi:10.1038/s41372-025-02390-2)
Supplement: Supplementary file 1 — Supplemental Table [file 41372_2025_2390_MOESM1_ESM.docx]

| Variables | Categories | | |
| --- | --- | --- | --- |
|  | 0 | 1 | 2 |
| Birth weight (grams) | < 580 | 580-870 | >870 |
| Gestational age (weeks) | <25 | 25-27 | >27 |
| Maternal platelets | <167 | 167-259 | >259 |
| Apgar scores | <1 | 1-5 | ≥5 |
| Blood gas  pH  pCO_2_  pO_2_ | <7.2  <41  <49 | 7.2-7.3  41-55  49-79 | >7.3  >55  >79 |
| 0-12 hours |  |  |  |
| FiO_2_  Average  Low  High | <0.28  0.21  <0.3 | 0.28-0.505  0.22-0.28  0.3-0.71 | >0.505  >0.28  >0.71 |
| Hematocrit | <37.3 | 37.3-46 | >46 |
| Heart rate (bpm)  Average  Low  High | <141  <126  <158 | 141-158  126-142  158-174 | >158  >142  >174 |
| Mean arterial pressure  Average  Low  High | <28  <22  <33 | 28-39.5  22-29  33-48 | >39.5  >29  >48 |

Supplemental Table. Variables categorized for the artificial intelligence model.
